# The Role of Sugar Transporter CsSWEET7a in Apoplasmic Phloem Unloading in Receptacle and Nectary During Cucumber Anthesis

**DOI:** 10.3389/fpls.2021.758526

**Published:** 2022-01-31

**Authors:** Yaxin Li, Huan Liu, Xuehui Yao, Lulu Sun, Xiaolei Sui

**Affiliations:** Beijing Key Laboratory of Growth and Developmental Regulation for Protected Vegetable Crops, College of Horticulture, China Agricultural University, Beijing, China

**Keywords:** *Cucumis sativus* L., hexose transporter, flowering, phloem transport, pollinator reward, SWEET protein

## Abstract

During anthesis, there is an increased demand for carbohydrates due to pollen maturation and nectary secretion that warrants a systematic phloem unloading strategy for sugar partitioning. Sugar transporters are key components of the apoplasmic phloem unloading strategy and control the sugar flux needed for plant development. Currently, the phloem unloading strategy during anthesis has not been explored in cucumber, and the question of which sugar transporters are active during flower anthesis is poorly understood. In this study, a study utilizing the phloem-mobile symplasmic tracer carboxyfluorescein (CF) suggested that the phloem unloading was symplasmically isolated in the receptacle and nectary of cucumber flowers at anthesis. We also identified a hexose transporter that is highly expressed in cucumber flower, Sugar Will Eventually be Exported Transporter 7a (SWEET7a). *CsSWEET7a* was mainly expressed in receptacle and nectary tissues in both male and female flowers, where its expression level increased rapidly right before anthesis. At anthesis, the CsSWEET7a protein was specifically localized to the phloem region of the receptacle and nectary, indicating that CsSWEET7a may function in the apoplasmic phloem unloading during flower anthesis. Although cucumber mainly transports raffinose family oligosaccharides (RFOs) in the phloem, sucrose, glucose, and fructose are the major sugars in the flower receptacle and the nectary as well as in nectar at anthesis. In addition, the transcript levels of genes encoding soluble sugar hydrolases (α*-galactosidase*, *sucrose synthase*, *cytoplasmic invertase*, and *cell wall invertase*) were correlated with that of *CsSWEET7a*. These results indicated that CsSWEET7a may be involved in sugar partitioning as an exporter in the phloem of the receptacle and nectary to supply carbohydrates for flower anthesis and nectar secretion in cucumber.

## Introduction

In flowering plants, the reproductive tissues require large amounts of carbohydrates for pollen maturation, nectar secretion, pollen tube generation, and seed initiation (Borghi and Fernie, 2017). Flower petals and sepals have limited capacity for photosynthesis when they are green, and this capacity further decreases before anthesis as the petal color changes (Müller et al., 2010). As a sink organ, the flower relies heavily on the sugar produced in leaves as the energy for its development. The sugar produced in the source leaf is transferred to a sink organ through three main steps: phloem loading (Zhang and Turgeon, 2018), phloem long-distance transport (Jensen, 2018), and phloem unloading (Milne et al., 2018). During phloem unloading, sugar is unloaded from the sieve element/companion cell (SE/CC) complex into sink tissues *via* one of the two pathways: flowing into phloem parenchyma cells through plasmodesmata (symplasmic unloading) or transport through the apoplasm mediated by sugar transporters (apoplasmic unloading) (Oparka, 1990).

Within a flower, different tissues may use different unloading strategies. For example, pollen grains and pollen tubes are symplasmically isolated from surrounding tissues and utilize an apoplasmic unloading strategy (Borghi and Fernie, 2017). In *Arabidopsis*, carbohydrate movement into the anther *via* the filament uses a symplasmic unloading strategy (Imlau et al., 1999). Similarly, phloem unloading in the *Arabidopsis* petal is symplasmic (Imlau et al., 1999). However, it is not clear which phloem unloading strategies are utilized in the nectary and receptacle. In many plant species, the nectary aids reproduction through attracting pollinators by secreting nectar. The receptacle is often enlarged to support the flower and hold all flower tissues together. In cucumber (*Cucumis sativus* L.), the nectary in male flowers is button-like in appearance, usually three-lobed, and lies on the receptacle (Collison and Martin, 1975). The nectary in the female cucumber flower initiates at the junction between the base of the style and the receptacle before forming a ring-shaped structure (Bai et al., 2004). As non-photosynthetic organs, most of the nectary, as well as the secreted nectar, are dependent on phloem-derived sugars from sources (Roy et al., 2017). Phloem is reported to be the most common vascular tissue in nectary and the sugar content can reach up to 50% in phloem-rich nectary, while the sugar concentration can be as low as 8% in xylem dominant nectary (Frey-Wyssling, 1955; Roy et al., 2017). In *Arabidopsis* (Lin et al., 2014) and squash (Solhaug et al., 2019a), several steps are important for nectar secretion, including starch accumulation at the early developmental stage of nectary and subsequent starch degradation pre-anthesis, sucrose synthesis, and sucrose export. In addition, the direct transport of phloem sugar, without prior storage as starch, could also play an important role in the generation of squash nectar (Solhaug et al., 2019a). Thus, it is important to understand how carbohydrates are unloaded from phloem in the receptacle and nectary. As a model plant of unisexual floral development (Gu et al., 2011), cucumber offers a great opportunity to streamline the study of carbohydrate partitioning in flowers.

During apoplasmic unloading, transporters move sugars across membranes, with an exporter taking sugar from the SE/CC into the apoplasmic space and an energy-dependent importer taking sugar into phloem parenchyma cells (Milne et al., 2018). Sucrose transporter (SUT) and monosaccharide transporters [e.g., sugar transport protein (STP)] were reported to function at the latter step (Büttner, 2010; Borghi and Fernie, 2017), while a sugar exporter (functioning at the first step) has not been reported in flowers. Cucumber Sugar Will Eventually be Exported Transporter 7a (SWEET7a) is known to localize the companion cells in fruit vascular bundles and to export hexose to the apoplasmic space to stimulate sugar unloading in fruit (Li et al., 2021). In *Arabidopsis* pollen, AtSWEET8 and AtSWEET13 are involved in pollen maturation, and an aborted silique phenotype was observed in *sweet8* single mutant (Guan et al., 2008), while this phenotype was more severe in *sweet8;13* double mutant (Sun et al., 2013). AtSWEET9 is specifically expressed in nectary parenchyma cells and functions together with cell wall invertase (CWINV) and sucrose phosphate synthases (SPS) in nectary secretion (Lin et al., 2014). Similarly, SWEET9 homologs are involved in nectary secretion in *Brassica*, *Nicotiana*, and *Petunia hybrida* (Ge et al., 2000; Lin et al., 2014). Additionally, several SWEET homologs are expressed in flowers of *Arabidopsis* (Lin et al., 2014), cucumber (Li et al., 2017), and *Jasminum sambac* (Wang et al., 2019). Besides, SWEETs function as a uniporter, which facilitates sugar transport along substrate gradient. Specifically, Clade I/II SWEETs mainly transport hexoses and Clade III SWEETs transport sucrose, while Clade IV SWEETs are tonoplast-localized hexose transporters (Chen et al., 2015a). All SWEETs identified so far function as low-affinity sugar transporters (with measured Km values in mM range) (Chen et al., 2015b), suggesting that they tend to have important roles in regions where sugars are abundant, such as the study of AtSWEET11,12,15 in seeds (Chen et al., 2015b) and CsSWEET7a in fruit (Li et al., 2021). Taken together, we hypothesized that SWEET transporters may be involved in phloem unloading in the receptacle and nectary of cucumber flowers during anthesis.

In this study, we found that phloem unloading in cucumber receptacle and nectary at anthesis is apoplasmic. Hexose transporter *CsSWEET7a* was highly expressed in nectary and receptacle in both male and female cucumber flowers, and its expression level increased as anthesis progressed. The CsSWEET7a protein is specifically localized in the phloem of nectary and receptacle during anthesis, indicating a possible role in sugar phloem unloading at these regions. Additionally, the transcript levels of genes encoding soluble sugar hydrolases (α*-galactosidase*, *sucrose synthase*, *cytoplasmic invertase*, and *CWINV*) were correlated with that of *CsSWEET7a*, suggesting a potential synergic relationship between *CsSWEET7a* and sugar hydrolases during apoplasmic phloem unloading in cucumber nectary and receptacle at anthesis.

## Materials and Methods

### Plant Material and Growth Conditions

Cucumber (*Cucumis sativus* L. “Xintaimici”) plants were grown in a greenhouse under natural light conditions from late February to July at China Agricultural University in Beijing. In brief, cucumber seeds were germinated for 2 days at 28°C in dark in a growth chamber before being transferred to the greenhouse under standard conditions. Water management and pest control were performed as needed.

### Carboxyfluorescein Diacetate Labeling

Carboxyfluorescein diacetate (CFDA) (Sigma-Aldrich, Shanghai, China) was first dissolved in acetone and then diluted to 0.5 mg ml^–1^ with water. The leaf in the same node as a male or female flower was chosen for CFDA labeling according to the study by Sui et al. (2018) with minor adjustment. The upper leaf epidermis was abraded with fine sandpaper. Then, 200 μl CFDA solution was applied, and the leaf was covered with plastic wrap to prevent evaporation. After 6-h labeling, the male and female flowers were sampled. A hand-section of nectary and receptacle samples was examined by confocal laser scanning microscopy (CLSM) (Confocal, Tokyo, Japan).

### Spatial and Temporal Expression Analysis by Reverse Transcription-Quantitative PCR

For temporal expression analysis, male and female flowers were sampled at different developmental stages according to the study by Bai et al. (2004) at zeitgeber time (ZT) 4. The whole flower was sampled and dissected under a stereomicroscope (LEICA, S8APO, Germany) for receptacle and nectary samples. Three biological replicates were prepared. For gene expression analysis in different tissues, including root, stem, leaf, male and female flowers, and ovary/fruit, the samples were harvested at ZT4 of the 2-month-old cucumber plant at anthesis.

Total RNA from different tissues was extracted using the RNeasy Plant Kit (Huayueyang, Beijing, China) according to the protocol of the manufacturer. Reverse transcription was carried out using the FastQuant RT Kit (with gDNase; Tiangen, Beijing, China). Gene expression analyses were performed by reverse transcription-quantitative PCR (RT-qPCR) with the SYBR green detection protocol (TaKaRa, Japan) on an ABI 7500 Real-Time PCR Detection System (Bio-Rad, United States). The relative expression level was normalized to the housekeeping gene *Tubulin* using the 2^–ΔΔCT^ method (Livak and Schmittgen, 2001). Primers used in this study are as shown in Supplementary Table 1.

### Immunohistochemical Localization and Microscopy

*CsSWEET7a* primary antibody was described by Li et al. (2021). An alkaline phosphatase (AP)-labeled Goat Anti-Rabbit IgG was used as the secondary antibody. For immunohistochemical analyses, the receptacle and nectary samples from male and female flowers were harvested on the day of anthesis and dissected under a stereomicroscope. All samples were fixed in formaldehyde-acetic acid-ethanol (FAA) solution, followed by series dehydration, and were embedded in the wax as previously described (Wang et al., 2014). The immunohistochemical localization analysis was performed according to the study by Li et al. (2021). After the immunohistochemical reaction, samples were applied with 80% (v/v) glycerin and coverslip to keep moisture. Immunohistochemical signals were imaged under an Olympus microscope (BX53, Japan). Immunohistochemical signals of the whole receptacle and nectary tissues were observed under the 4 × objective, and the zoom-in images of vascular inside receptacle and nectary were taken under 10× or 20× objective.

### Carbohydrate Extraction and Analysis

For sugar content measurement, around 0.2 g (fresh weight) receptacle, 0.05 g (fresh weight) nectary, and 30 μl nectar were sampled at ZT4 from pools of 10 flowers. Three biological replicates were taken. The receptacle and nectary were sampled under a stereomicroscope (LEICA, S8APO, Germany), and the nectar was sampled with a 10-μl pipette. Soluble sugar was extracted according to Ma et al. (2019a) with minor adjustments. In brief, the receptacle and nectary were homogenized in 200 μl 80% (v/v) ethanol in a 1.5-ml tube with a hand-held homogenizer. To the homogenate, 800 μl of 80% (v/v) ethanol was added. Samples were extracted under 80°C for 30 min. Samples were centrifuged at 12,000 × *g* for 10 min. The supernatant was transferred to a new 5-ml tube. Extractions were repeated twice, with the three supernatants combined. Supernatants were evaporated to dryness at 40°C, dissolved in 500 μl Mili-Q water, and filtered through a 0.22-mm nylon filter membrane. Nectar samples were diluted five times and filtered through a 0.22-mm nylon filter membrane. All samples were analyzed by High-Performance Liquid Chromatography (HPLC, Dao Jin RID-20A, Japan). The Shodex Asahipak NH2P-50 4E column was used as the separation column, and 70% (v/v) acetonitrile was used as the mobile phase. The flow rate was 1 ml/min, and the column temperature was 40°C.

For starch staining, medial longitudinal anatomy of male and female flower tissues was immersed in 10% I-KI staining solution for 30 min, then washed with water 2–3 times to stop the reaction, and removed the extra staining solution. The starch accumulated tissues were stained dark brown. Images were taken under a stereomicroscope (LEICA, S8APO, Germany).

### Statistical Analysis

Statistical analyses in this study were performed using one−way ANOVA followed by multiple comparisons using Fisher’s least significant difference method (*P−*value < 0.05) using Origin 2021b (OriginLab, Northampton, MA, United States).

## Results

### Phloem Unloading in Flower Nectary and Receptacle Followed an Apoplasmic Pathway at Anthesis

**FIGURE 1:**
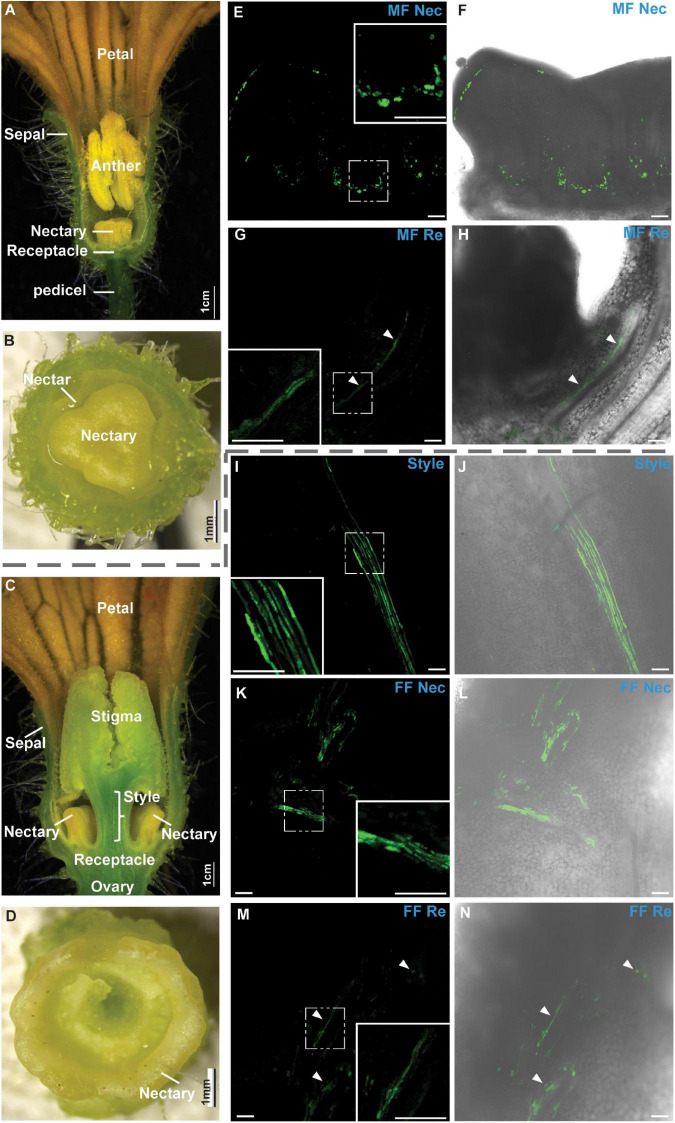
Microscopic images of cucumber flowers at anthesis. **(A–D)** Medial longitudinal **(A,C)** and transverse anatomy images **(B,D)** of male **(A,B)** and female flowers **(C,D)** at anthesis. **(E–N)** Confocal laser scanning microscopy (CLSM) of carboxyfluorescein (CF) unloading from the phloem was shown in male **(E–H)** and female flowers **(I–N)**. CF signal was labeled in green in male and female flowers 6 h after feeding carboxyfluorescein diacetate (CFDA) at the leaf. CF signal in nectary (Nec) **(E,F)** and receptacle (Re) **(G,H)** of male flower (MF); in style **(I,J)**, nectary **(K,L)**, and receptacle **(M,N)** of the female flower (FF), respectively. The boxed insets in panels **(E,G,I,K,M)** were the close-up images. Bars in panels **(E–N)** were 100 μm. Bars in the close-up images were 50 μm. White arrows indicate the CF signal.

We first observed the anatomy of male and female cucumber flowers at anthesis (Figures 1A–D). Images of the medial longitudinal anatomy of male flowers showed structures including sepal, petal, pedicel, receptacle, nectary, and anther (Figure 1A; the filament was not shown in this figure). The receptacle was located at the bottom, and the nectary was on the top center of the receptacle (Figure 1A, Bai et al., 2004). The transverse view of a male flower showed that the nectary initiated at the center of the receptacle and that the nectar was stored in between the nectary and receptacle (Figure 1B). Images of the medial longitudinal anatomy of female flowers showed structures including sepal, petal, ovary, receptacle, nectary, style, and stigma (Figure 1C). The receptacle connected the style and the ovary, and the nectary initiated at the junctions between the style and the receptacle before developing into a ring shape structure (Figures 1C,D).

The membrane-permeable non-fluorescent dye CFDA, which can be deacetylated into the fluorescent CF, a membrane-impermeable fluorescent dye, was used to investigate the phloem unloading pathways in cucumber receptacle and nectary at anthesis. CF has been successfully used in multiple species and tissues for phloem transport and unloading pattern analysis (Haupt et al., 2001; Viola et al., 2001; Hu et al., 2011; Palmer et al., 2015; Sui et al., 2018). In this study, CFDA was applied to the leaf, and the CF signal was sought in the nearest male or female flower. If the plants use a symplasmic phloem unloading strategy, the CF will expand from phloem to the parenchyma cell through plasmodesmata, and a relatively uniform signal should be observed all over the tissues; if the plants undertake an apoplasmic unloading strategy, the CF will be found only in the phloem region and not dissipated to the surrounding parenchyma cells. In male flowers, the CF signal was confined in vascular regions without apparent diffusion to the surrounding tissues in the nectary (Figures 1E,F) or receptacle (Figures 1G,H). In female flowers, the CF signal was always confined to the phloem strands along the phloem pathway in the vascular bundles without apparent diffusion to the surrounding tissues in the style (Figures 1I,J), nectary (Figures 1K,L), or receptacle (Figures 1M,N). This indicated that phloem unloading in these tissues at anthesis was apoplasmic, and sugar transporters were probably required during this process.

### *CsSWEET7a* Was Highly Expressed in Flower Receptacle and Nectary During Anthesis

A total of 17 SWEET genes were identified in the cucumber genome (Li et al., 2017). We surveyed the expression level of all cucumber SWEET genes in male and female flowers at anthesis using an RNA-seq dataset (PRJNA80169) from cucumber.^1^ *CsSWEET1*, *CsSWEET7a*, *CsSWEET9*, and *CsSWEET17a* showed relatively higher expression levels than the other *SWEETs* in flowers at anthesis, with *CsSWEET7a* showing the highest expression among Clade I/II SWEETs (Supplementary Figure 1A). *CsSWEET9* of Clade III is a homolog of *Arabidopsis SWEET9*, which is expressed in nectary epidermal cells to move sucrose into nectar for secretion (Lin et al., 2014). In our previous study, CsSWEET17a of Clade IV was localized on the tonoplast (Li et al., 2017). *CsSWEET7a* was highly expressed in sink tissues (e.g., flower, root, and fruit), especially in flowers (Li et al., 2017). CsSWEET7a was reported to be localized on the plasma membrane of companion cells in cucumber fruit vasculature (Li et al., 2021), which suggests that CsSWEET7a is a promising candidate for phloem unloading in flowers. We further tested the expression pattern of *CsSWEET7a* at different developmental stages of male flowers [Supplementary Figure 1B; stage division as described by Bai et al. (2004) and Sun et al. (2019)]. In brief, at stage 9, microsporocytes initiate within a 1.5–2 mm length flower bud; at stage 10, anthers meiosis and nectary tissues initiate within a 3–4 mm length flower bud; at stage 11, uninuclear pollen appears, and nectary tissues form a ring within a 4–10 mm length flower bud; at stage 12, mature pollen forms, and nectary tissues fully develop in a 10–20 mm length flower bud; at stage 13, the anthesis is initiated. Our results showed that the expression level of *CsSWEET7a* slowly increased from stage 9 to stage 11 before rapidly increasing from stage 11 and peaking at anthesis (Supplementary Figure 1B), when flower tissues matured and prepared for anthesis, e.g., uninuclear pollen developed to mature pollen, and nectary tissues are fully developed in male flowers (Bai et al., 2004). In our previous study, the sugar level in male cucumber flowers increased from stage 9 to stage 12 (Sun et al., 2019). Thus, the increased expression level of *CsSWEET7a* in male flowers correlates well with the accumulated sugar levels and suggests that it may participate in sugar partitioning during this process.

**FIGURE 2:**
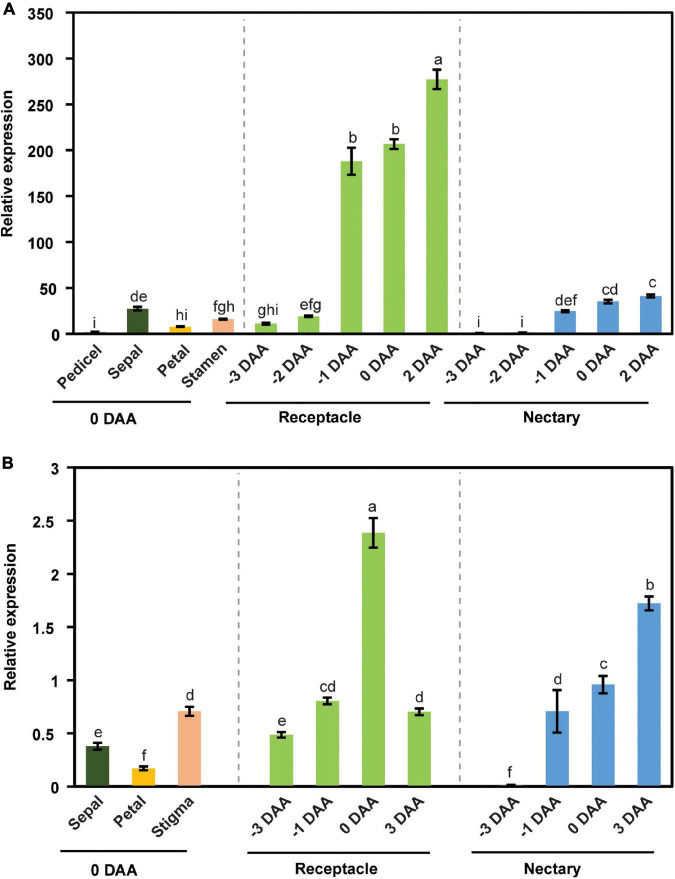
The expression pattern of *CsSWEET7a* in male **(A)** and female flowers **(B)**. DAA, days after anthesis; −3, −2, −1 DAA, being 3, 2, and 1 day(s) before anthesis, respectively; 0 DAA, the day of anthesis; and 2 and 3 DAA, being 2 and 3 days after anthesis, respectively. Mean values ± SE of three independent biological replicates were shown. Statistical analyses were performed using one-way ANOVA followed by multiple comparisons using Fisher’s LSD method (*P*-value < 0.05).

We further examined the expression pattern of *CsSWEET7a* in different tissues in male and female flowers across flower development. *CsSWEET7a* was highly expressed in receptacle and nectary in both male (Figure 2A) and female flowers (Figure 2B). As the *CsSWEET7a* expression level increased at stage 11 in male flowers, we started to harvest receptacle and nectary samples at 3 days before anthesis (stage 11 to stage 12 samples). The expression level of *CsSWEET7a* increased from 3 days before anthesis to the day of anthesis or even 2–3 days after anthesis in receptacle and nectary of both male (Figure 2A) and female flowers (Figure 2B). This indicated that CsSWEET7a may function in receptacle and nectary regions for sugar partitioning during anthesis when the demand for sugar peaks.

### CsSWEET7a Protein Was Specifically Localized in the Phloem Region in Cucumber Flowers

**FIGURE 3:**
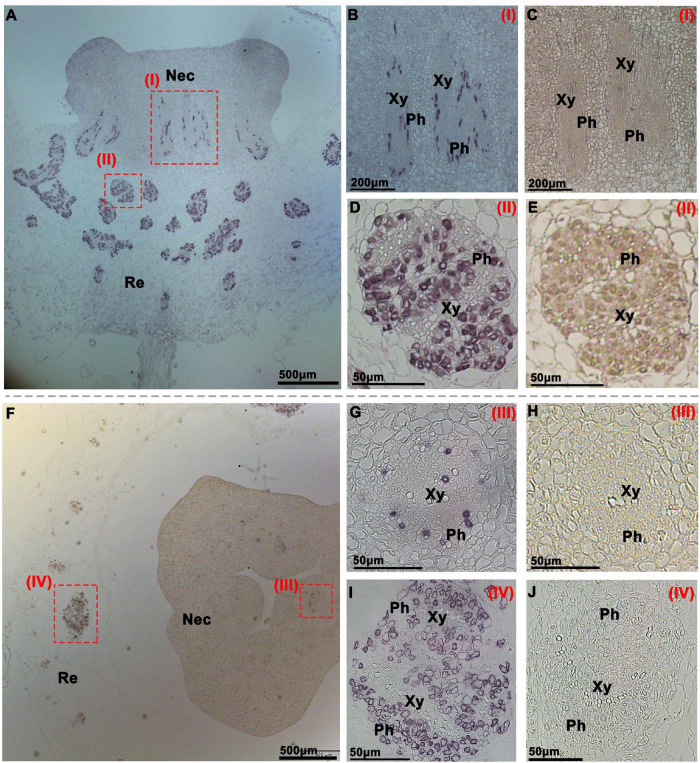
Immunohistochemical localization of *CsSWEET7a* in male cucumber flowers. Longitudinal **(A–E)** and transverse sections **(F–J)** of male flowers with alkaline phosphatase (AP) as the second antibody. **(B,D,G,I)** The close-up of the boxes in panels **(A,F)**, respectively. **(C,E,H,J)** Sections incubated with pre-immune serum as a control. The Roman numerals in red at the top-right corner **(B–E,G–J)** correspond to the fields in panels **(A,F)**. Nec, nectary; Re, receptacle; Ph, phloem; Xy, xylem.

**FIGURE 4:**
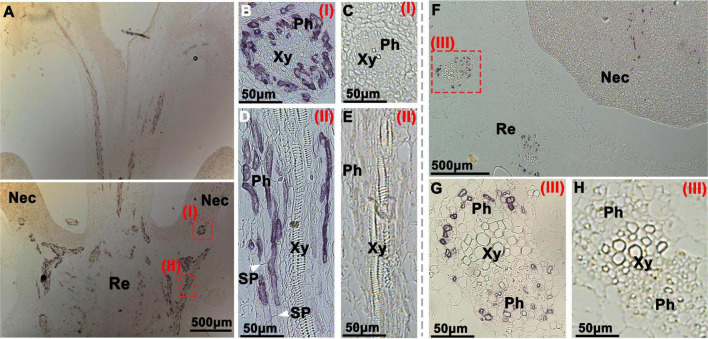
Immunohistochemical localization of CsSWEET7a in female cucumber flowers. Longitudinal **(A–E)** and transverse sections **(F–H)** of female flowers with AP as the second antibody. **(B,D,G)** The close-up of the boxes in panels **(A,F)**, respectively. The white arrows in panel **(D)** indicate the sieve plate. **(C,E,H)** Sections were incubated with pre-immune serum as a control. Images in panels **(G,H)** were rotated 90°clockwise. The Roman numerals in red at the top-right corner **(B–E,G,H)** correspond to those fields in panels **(A,F)**. Nec, nectary; Re, receptacle; Ph, phloem; Xy, xylem; SP, sieve plate.

To investigate the function of CsSWEET7a in the receptacle and nectary of male and female flowers during anthesis, we analyzed CsSWEET7a protein localization by immunohistochemical staining (Figures 3, 4). A polyclonal antibody was generated using two CsSWEET7a-specific peptides as described by Li et al. (2021). The receptacle and nectary regions of both male and female flowers at anthesis were fixed in wax for sectioning. A strong and specific immunohistochemical signal from the CsSWEET7a protein was observed in vascular tissues of receptacle and nectary both in longitudinal (Figure 3A) and transverse sections (Figure 3F) in male flowers. In close-up views, we found that CsSWEET7a was highly expressed in the phloem region of the nectary (Figures 3B,G) and receptacle (Figures 3D,I) and that no signal was observed in sections incubated with pre-immune serum (Figures 3C,E,H,J). Interestingly, the vascular density was higher in the receptacle than in the nectary, and more CsSWEET7a proteins were detected in the phloem tissue of the receptacle region than in the nectary region (Figures 3A,F). This indicates that the receptacle, which connects the pedicle and nectary, could function as a hub for carbohydrate partitioning in male flowers.

In the longitudinal sections of female flowers, a large number of vascular signatures showed immunohistochemical signals from the CsSWEET7a protein, which were strongly and specifically observed in the receptacle (Figure 4A), similar to those in the male flowers. In close-up views of the receptacle, CsSWEET7a was localized to the cells at both sides of sieve elements, most likely the companion cells (Figure 4D). This agrees with the CsSWEET7a protein localization in fruit vascular tissues (Li et al., 2021). CsSWEET7a is also localized to the vascular tissues in the nectary (Figures 4A,B) and style (Figure 4A). In the transverse view of female flowers, CsSWEET7a protein signal was observed in vascular tissues in the receptacle as well as in nectary (Figures 4F,G), like what we observed in male flowers. No signals were observed in sections incubated with pre-immune serum (Figures 4C,E,H). Overall, CsSWEET7a protein was specifically expressed in phloem tissues in receptacle and nectary in both male and female cucumber flowers at anthesis.

### Sucrose, Glucose, and Fructose Were the Major Sugars in Flower Receptacle and Nectary at Anthesis

**FIGURE 5:**
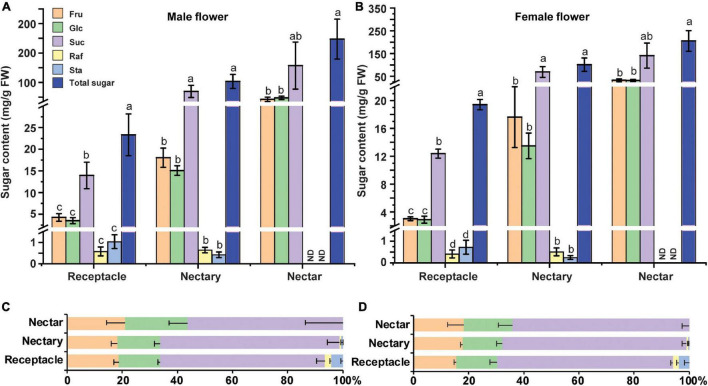
Sugar content in male and female cucumber flowers at anthesis. Sugar content **(A,B)** and sugar distribution (%) **(C,D)** in receptacle, nectary, and nectar of male **(A,C)** and female flowers **(B,D)** at anthesis. Fru, fructose; Glc, glucose; Suc, sucrose; Raf, raffinose; Sta, stachyose; FW, fresh weight. ND, not detected. For each sample in this figure, ten flowers were collected and combined as a sample. Mean values ± SE of three independent biological replicates were shown. Statistical analyses were performed using one-way ANOVA followed by multiple comparisons using the Fisher’s LSD method (*P*-value < 0.05) in each tissue.

The soluble sugar level in cucumber male flowers was reported to increase from stage 9 to stage 12 (Sun et al., 2019). Specifically, sucrose, glucose, and fructose are the major soluble sugar components, with a small amount of raffinose and stachyose in cucumber male flowers (Sun et al., 2019). To further investigate the sugar compositions in the receptacle, nectary, and nectar, we sampled these tissues at anthesis and extracted sugar for HPLC analysis. The results showed that nectar has the highest sugar level, followed by nectary and receptacle in both male (Figure 5A) and female flowers (Figure 5B). Moreover, sucrose, glucose, and fructose were the major soluble sugars, with only small amounts of raffinose and stachyose detected (Figures 5A,B). In the receptacle, nectary, and nectar of both male and female flowers, the majority of the sugar was sucrose (56–65%), followed by fructose (18–21%) and glucose (15–23%) (Figures 5C,D). Although the sugar compositions in the receptacle and nectary are sucrose-dominant, more than 30% hexoses are still present in the receptacle and nectary. Thus, CsSWEET7a is likely involved in the hexoses unloading, especially in the receptacle of both male and female flowers, given its strong phloem localization signals and gene expression pattern. Additionally, it has been reported that phloem-derived sugar was stored as starch in nectary before anthesis in squash (*Cucurbita pepo*), and the starch will be hydrolyzed to produce sugar for rapid energy supply during anthesis (Solhaug et al., 2019b). Thus, we examined the starch accumulation before and during anthesis in cucumber flowers (from −3 DAA to 0 DAA) (Supplementary Figure 3). Clear starch accumulation was observed 1–3 days before anthesis (Supplementary Figures 3A,B,D,E) in both male and female flowers, especially in the nectary, but almost all the starch was hydrolyzed at anthesis (Supplementary Figures 3C,F).

### Raffinose Family Oligosaccharide Metabolism Genes Are Regulated During the Anthesis Stage in the Receptacle and Nectary

**FIGURE 6:**
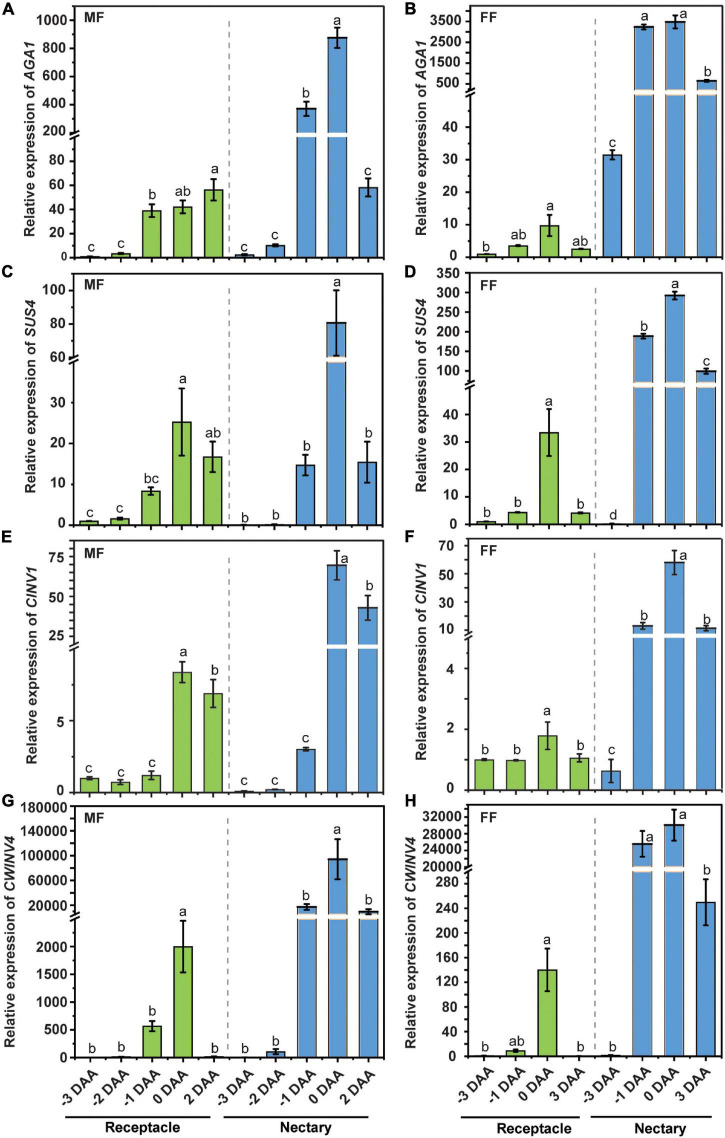
The expression pattern of sugar metabolism enzymes in cucumber receptacle and nectary. The relative expression level of cucumber *AGA1*, *SUS4*, *CINV1*, and *CWINV4* in male **(A,C,E,G)** and female flowers **(B,D,F,H)**. DAA, days after anthesis; −3, −2, −1 DAA, being 3, 2, and 1 day(s) before anthesis, respectively; 0 DAA, the day of anthesis; and 2 and 3 DAA, being 2 and 3 days after anthesis, respectively. Mean values ± SE of three independent biological replicates were shown. Statistical analyses were performed using one-way ANOVA followed by multiple comparisons using the Fisher’s LSD method (*P*-value < 0.05) in each tissue. Gene IDs: *AGA1* (Csa4G631570), *alkaline a-galactosidase 1*; *SUS4* (Csa5G322500), *sucrose synthase 4*; *CINV1* (Csa5G615240), *cytosol invertase 1*; *CWINV4* (Csa2G351670), *cell wall invertase 4*.

Cucumber is a typical raffinose family oligosaccharide (RFO)-transporting plant, and in other words, the majority of the transporting sugar in phloem sap is stachyose and raffinose, with a small amount of sucrose. The stachyose/raffinose was first hydrolyzed by α-galactosidase to sucrose, which was further catalyzed by sucrose synthase (SUS) and/or invertase (INV) to produce hexoses, and the resulting hexoses can be exported by CsWEET7a into apoplasmic space in the cucumber fruit (Li et al., 2021). Similarly, there is only a trace amount of stachyose/raffinose in the cucumber receptacle, nectary, and nectar (Figure 5). Moreover, it was found that the transcript coding for many RFO/sucrose catabolism-related enzymes, such as *alkaline* α*-galactosidase 1* (*AGA1*), *sucrose synthase 4* (*SUS4*), *cytoplasmic invertase 1* (*CINV1*), and *cell wall invertase 4 (CWINV4)*, were highly expressed in both male and female flowers at anthesis compared with other tissues, including root, stem, and leaf (Supplementary Figure 2), indicating that they are most likely to be involved in active sugar hydrolysis during this period. To investigate if a similar sugar unloading strategy was undertaken at receptacle and nectary compared with that in the fruit, we further tested the expression pattern of *AGA1*, *SUS4*, *CINV1*, and *CWINV4* in receptacle and nectary at various cucumber flower developmental stages. In male (Figures 6A,C,E,G) and female flowers (Figures 6B,D,F,H), the expression levels of *AGA1*, *SUS4*, *CINV1*, and *CWINV4* peaked at anthesis in both receptacle and nectary compared with earlier developmental stages. Overall, these four sugar metabolic genes showed a similar expression pattern as *CsSWEET7a* in either receptacle or nectary of male and female flowers during anthesis (Figure 2), suggesting potential cooperation between sugar catabolism enzymes and sugar transporter CsSWEET7a in apoplasmic phloem unloading in cucumber receptacle and nectary.

## Discussion

### The Significance of Apoplasmic Phloem Unloading in Flower Receptacle and Nectary at Anthesis

Plants use different unloading pathways depending on different types of sink organs, developmental stages, or changes in the environment (Milne et al., 2018; Ma et al., 2019b). Symplasmic unloading is common in meristems and in starch- or oil-storing sink organs, while apoplasmic unloading is often used in cell expansion zones and organs that accumulate soluble sugars (Oparka, 1990; Ma et al., 2019b). The use of a suitable unloading strategy can help plants save energy and adapt to environmental changes. To our knowledge, this study is the first to report that an apoplasmic phloem unloading strategy is used in the receptacle and the nectary in male and female cucumber flowers at anthesis (Figure 1). Compared with symplasmic unloading, which depends on both the sugar gradient (osmosis occurs along a concentration gradient) and the density of plasmodesmata, during apoplasmic unloading, sugars were exported from SE-CCs to apoplasmic space *via* an exporter, before taking up by an energy-dependent importer into phloem parenchyma cells against the concentration gradient. In flowers, a high rate of sugar import is needed during anthesis to meet the demands of pollen maturation, of nectar secretion (Borghi and Fernie, 2017), and of the increasing respiratory rate that raises the floral temperature for scent volatilization (Seymour, 1999). The soluble sugar level in male cucumber flowers increased from stage 9 to stage 11 and was maintained at the high level in stage 12 (Sun et al., 2019). If the receptacle and nectary parenchyma cells were connected to the SE/CC through a large number of plasmodesmata (symplasmic pathway), sugar might flow back symplasmically to the phloem through the connected plasmodesmata. The apoplasmic unloading mechanism seen in cucumber is common in horticultural plants, especially in the fruit (the harvestable product) where sugar can accumulate to high levels. For example, in grape berries, the phloem unloading shifts from symplasmic to apoplasmic during fruit maturation (Zhang et al., 2006). At the green fruit stage, symplasmic unloading is energy conservative. But at the mature stage, higher levels of soluble sugar accumulates in fruit to the point that the sugar content in fruit cells is higher than that in phloem sap, and sugar unloading shifts to the apoplasmic pathway (Zhang et al., 2006).

It was reported that before anthesis in squash, a species that also transports RFOs in its phloem, a massive amount of starch was stored in nectary and ready to be hydrolyzed the day before anthesis (Solhaug et al., 2019a). A similar starch accumulation pattern was observed in the cucumber nectary in our study (Supplementary Figure 3). Abundant starch was storied in nectary at 1–3 days before anthesis in both male and female cucumber flowers, but almost all the starch was hydrolyzed at anthesis. In *Arabidopsis*, which is a sucrose-transporting species, it might rely more on nectary starch degradation to produce nectar sugar rather than the import of phloem-derive sugars (Lin et al., 2014). But in squash, Solhaug et al. (2019a) estimated that ∼59% of the total sugar in the nectary/nectar system comes from starch, meanwhile, the imported sugar from the phloem makes up a substantial portion of total system sugar (∼41%), suggesting that phloem-derived sugar is important in nectar production at anthesis. Notably, cucumber nectary accumulates a high level of starch at pre-anthesis stages and thus maintains a constant sink status, negating a need for active apoplasmic unloading. We only carried a CF study to investigate the phloem unloading pathway at anthesis, thus the phloem unloading strategy at the early flower developmental stage in cucumber is still uncertain.

### Sugar Phloem Unloading in Cucumber Flowers During Anthesis Depends on Sugar Transporters and Sugar Metabolism Enzymes

Although many SWEET transporters are expressed at anthesis (Li et al., 2017; Wang et al., 2019), their specific expression pattern and functions in flowers remain unclear. In cucumber, the spatial and temporal expression analysis of *CsSWEET7a* by qRT-PCR and protein localization showed that CsSWEET7a might be involved in sugar partitioning in receptacle and nectary during anthesis. CsSWEET7a was previously reported to function as a hexose transporter in companion cells during fruit phloem unloading (Li et al., 2021). The *CsSWEET7a*-OE lines produced bigger fruit and flowers, while *CsSWEET7a*-RNAi lines had more photoassimilate trapped in the stem, resulting in smaller fruit and flowers (Li et al., 2021). In this study, CsSWEET7a protein was confirmed to be localized in the region of the phloem in the receptacle and nectary of both male and female flowers (Figures 3, 4). Therefore, CsSWEET7a could serve a similar function in the receptacle and nectary for phloem unloading, as reported in cucumber fruit. However, CsSWEET1, another plasma-membrane localized hexose transporter (Li et al., 2017), and CsSWEET9, a predicted sucrose transporter, also have a relatively high expression level in flowers (Supplementary Figure 1). We therefore cannot exclude their role in sugar phloem unloading.

**FIGURE 7:**
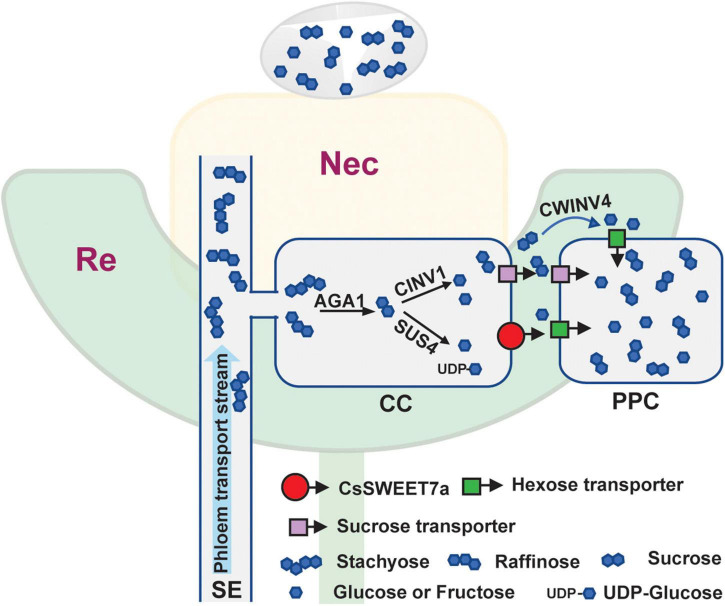
Simplified model of CsSWEET7a function during phloem unloading in cucumber receptacle and nectary. The top light-yellow color region indicates the nectary (Nec) tissue, and the bottom light-green color region is the receptacle (Re) tissue. The droplet above the nectary represents the secreted nectar. A similar phloem unloading process is proposed in receptacle and nectary of male and female flowers. Photosynthetic sugars are transported to flower receptacles and nectary tissues *via* the phloem. As an RFOs-transporting plant, the majority of sugar in the cucumber phloem transport stream [in sieve element (SE)] is stachyose and raffinose, with a small amount of sucrose detected. The stachyose and raffinose could be broken down by α-galactosidase 1 (AGA1) to sucrose, which has three possible routes to phloem parenchyma cell (PPC): (1) sucrose is hydrolyzed by sucrose synthase 4 (SUS4) and/or cytosol invertase 1 (CINV1) to hexoses in companion cell (CC), and the resulting hexoses are exported to apoplasmic space by hexose transporter, e.g., CsSWEET7a or other SWEETs (Clade I/II which transport hexoses), before loading into PPC by hexose transporter; (2) sucrose is exported from CC to apoplasmic space by sucrose transporter, followed by hydrolyzation *via* CWINV4 to hexoses, and hexoses are taken up to PPC by hexose transporter; (3) sucrose is exported to apoplasmic space by sucrose transporter and is taken up by sucrose transporter to PPC.

Cucumber mainly transports its sugars as RFOs, such as stachyose and raffinose, while sucrose, glucose, and fructose are primarily accumulated in cucumber young fruits (Hu et al., 2009) and flowers (Figure 5). Accordingly, the RFOs in the release phloem need to be broken down by AGA to sucrose. The sucrose is then hydrolyzed by SUS and/or INV to produce hexoses, which can be used for fruit development (Li et al., 2021). It suggests that cucumber sink tissues could adopt an apoplasmic phloem unloading strategy along with sugar deposition. In this study, an elevated expression of genes coding for sugar metabolism-related enzymes (Figure 6) as well as *CsSWEET7a* in both male and female flowers at anthesis (Figure 2) was observed, in support of their potential synergistic roles during RFO and sucrose hydrolyzation and phloem unloading at anthesis. Besides, a large amount of sucrose was presented in cucumber receptacle, nectary, and nectar, indicating that sucrose transporters were also required in sugar phloem unloading. It has been reported that CsSUT1 protein (sucrose transporter) is expressed in the phloem tissue in the receptacle of male cucumber flowers (Sun et al., 2019), which indicates that CsSUT1 may participate in apoplasmic unloading of sucrose in male flowers. Thus, we proposed a model (Figure 7) to illustrate the sugar phloem unloading strategy employed in the cucumber receptacle and nectary. The AGA1, hydrolyzes RFOs in the release phloem, and the resulting sucrose is broken-down to hexoses by SUS4 and/or by cytoplasmic invertase CINV1. CsSWEET7a may function in companion cells to export hexoses to apoplasmic space, where hexoses can be taken up by other hexose transporters into phloem parenchyma cells. Sucrose transporters (e.g., SUTs and Clade III SWEETs that transport sucrose) and CWINV4 may also be involved in the phloem unloading process as shown in Figure 7.

## Conclusion

We have provided evidence that sugar phloem unloading is symplasmically isolated in both receptacle and nectary of male and female cucumber flowers at anthesis, and the phloem-localized sugar transporter CsSWEET7a is most likely involved in this apoplasmic phloem unloading. A series of sugar metabolism enzymes including cucumber AGA1, SUS4, CINV1, and CWINV4 may have played a synergistic role in nectary and receptacle during this phloem unloading process. Our findings will provide valuable insights into the sugar partitioning strategy employed by plants to supply carbohydrates for flower anthesis and nectar secretion to reward pollinators.

## Data Availability Statement

The original contributions presented in the study are included in the article/Supplementary Material, further inquiries can be directed to the corresponding author.

## Author Contributions

XS and YL conceived the project and designed the experiments. YL, HL, and XY performed most of the experiments and analyzed the data. LS provided technical assistance to YL. YL and XS wrote the manuscript. XS agreed to serve as the author responsible for contact and ensures communication. All authors contributed to the manuscript and approved the submitted version.

## Conflict of Interest

The authors declare that the research was conducted in the absence of any commercial or financial relationships that could be construed as a potential conflict of interest.

## Publisher’s Note

All claims expressed in this article are solely those of the authors and do not necessarily represent those of their affiliated organizations, or those of the publisher, the editors and the reviewers. Any product that may be evaluated in this article, or claim that may be made by its manufacturer, is not guaranteed or endorsed by the publisher.

## Footnotes

1
http://cucurbitgenomics.org/rnaseq/home

## Supplementary Material

Supplementary Figure 1 Expression patterns of the *SWEET* gene family in cucumber flowers. **(A)** The expression pattern of the cucumber *SWEET* gene family in male (MF) and female flowers (FF). Data used in panel **(A)** came from RNA-seq data (PRJNA80169) in the cucumber database. **(B)** The relative expression level of *CsSWEET7a* from stage 9 to anthesis in male flowers. Mean values ± SE of three independent biological replicates were given. RPKM, Reads Per Kilobase per Million mapped reads.

Supplementary Figure 2 Expression pattern of genes coding for sugar metabolism-related enzymes in cucumber. Relative expression pattern of *AGA1* **(A)**, *SUS4* **(B)**, *CINV1* **(C)**, and *CWINV4* **(D)** in root, stem, leaf, male flower, female flower, and ovary at anthesis. *AGA1* (Csa4G631570), *alkaline a-galactosidase 1*; *SUS4* (Csa5G322500), *sucrose synthase 4*; *CINV1* (Csa5G615240), *cytosol invertase 1*; *CWINV4* (Csa2G351670), *cell wall invertase 4*. Mean values ± SD of three replicates were shown. Statistical analyses were performed using one-way ANOVA followed by multiple comparisons using Fisher’s LSD method (*P*-value < 0.05).

Supplementary Figure 3 Starch staining in nectary of male and female cucumber flowers. Different developmental stages of male **(A–C)** and female flowers **(D–F)**. **(A,D)** is 3 days before anthesis, **(B,E)** is 1 day before anthesis, and **(C,F)** is at the day of anthesis. The white arrows in panels **(D–F)** indicate nectary. DAA, days after anthesis; MF, male flower; FF, female flower; Nec, nectary.

Supplementary Table 1 Primers used in this study.
