# Supplementary figures and images for: The Role of Sugar Transporter CsSWEET7a in Apoplasmic Phloem Unloading in Receptacle and Nectary During Cucumber Anthesis
Source: Front Plant Sci. 2022 Jan 31;12:758526. doi: 10.3389/fpls.2021.758526 (PMC8841823; doi:10.3389/fpls.2021.758526)

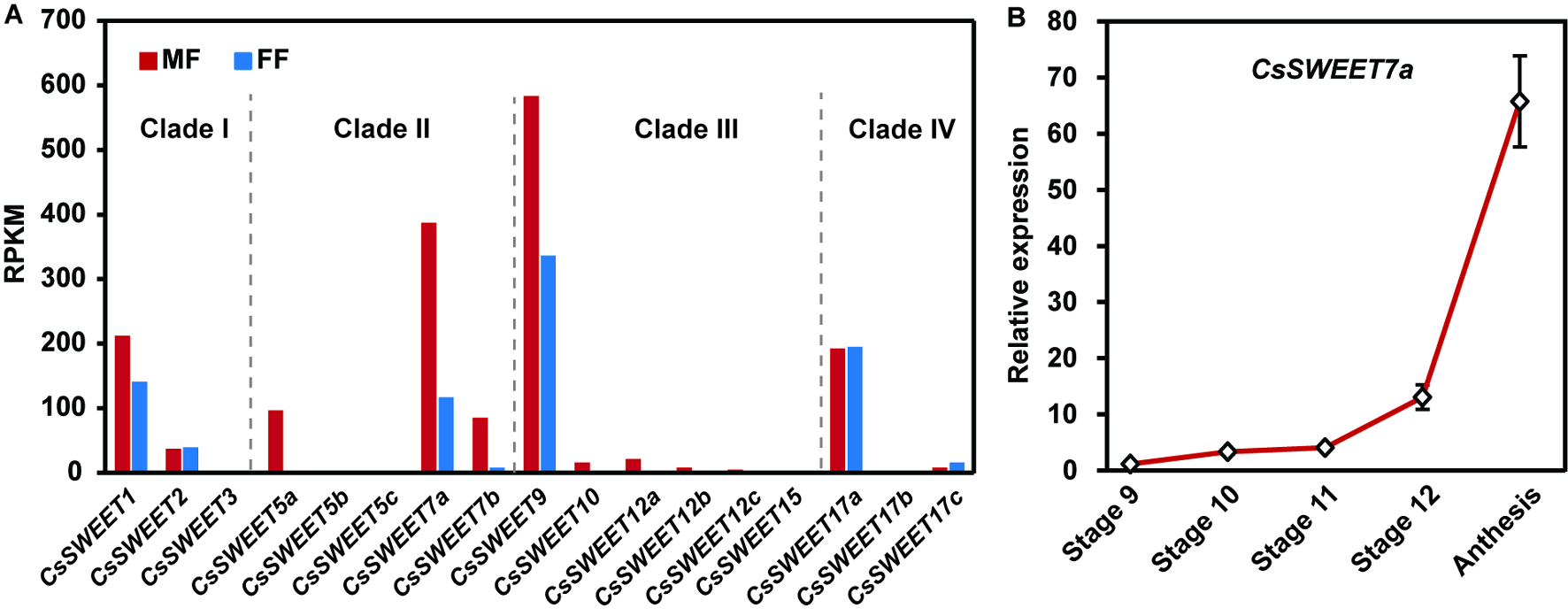

Supplement: Supplementary Figure 1 — Expression patterns of the SWEET gene family in cucumber flowers. (A) The expression pattern of the cucumber SWEET gene family in male (MF) and female flowers (FF). Data used in panel (A) came from RNA-seq data (PRJNA80169) in the cucumber database. (B) The relative expression level of CsSWEET7a from stage 9 to anthesis in male flowers. Mean values ± SE of three independent biological replicates were given. RPKM, Reads Per Kilobase per Million mapped reads. [file Image_1.TIF]

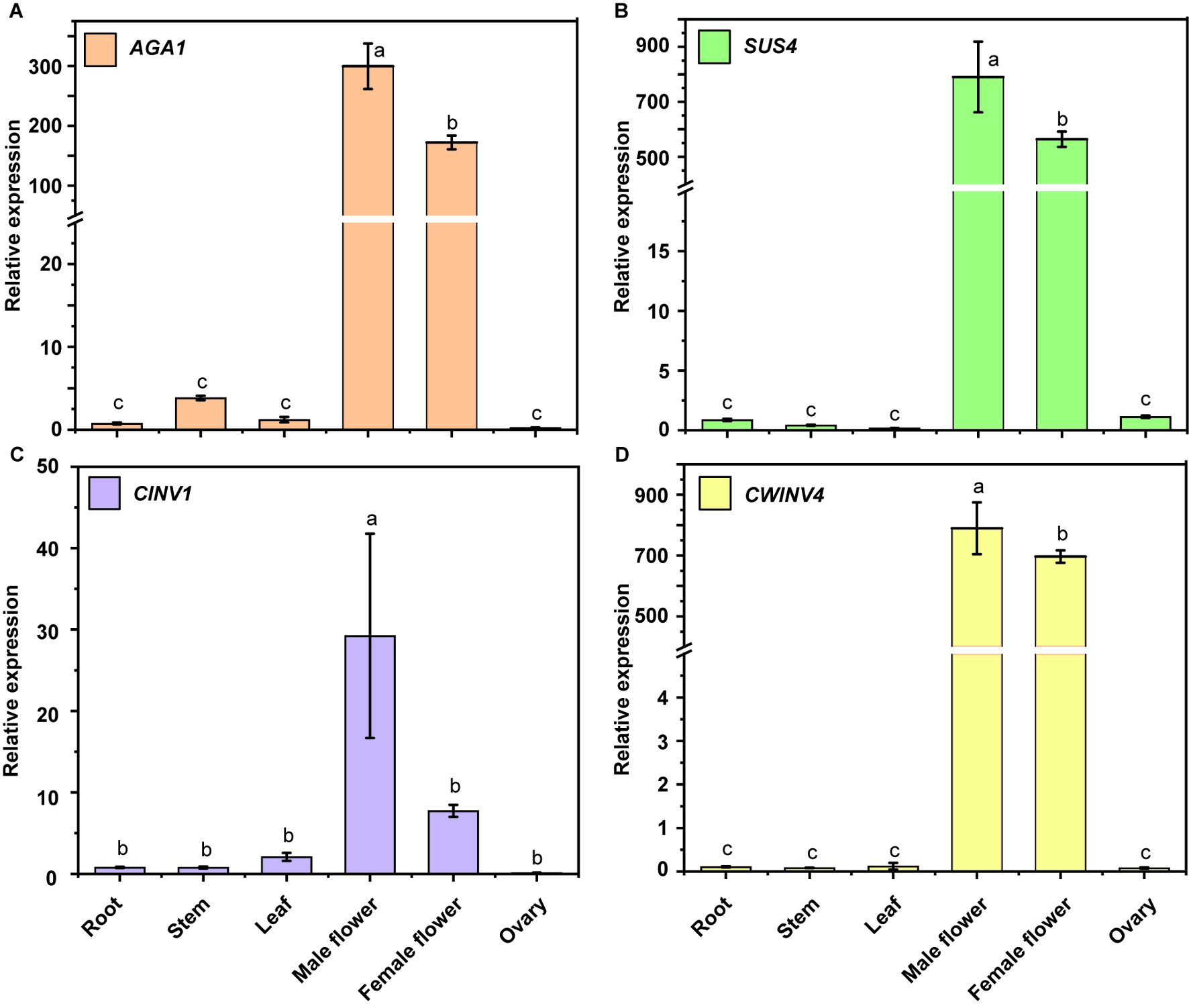

Supplement: Supplementary Figure 2 — Expression pattern of genes coding for sugar metabolism-related enzymes in cucumber. Relative expression pattern of AGA1 (A), SUS4 (B), CINV1 (C), and CWINV4 (D) in root, stem, leaf, male flower, female flower, and ovary at anthesis. AGA1 (Csa4G631570), alkaline a-galactosidase 1; SUS4 (Csa5G322500), sucrose synthase 4; CINV1 (Csa5G615240), cytosol invertase 1; CWINV4 (Csa2G351670), cell wall invertase 4. Mean values ± SD of three replicates were shown. Statistical analyses were performed using one-way ANOVA followed by multiple comparisons using Fisher’s LSD method (P-value < 0.05). [file Image_2.TIF]

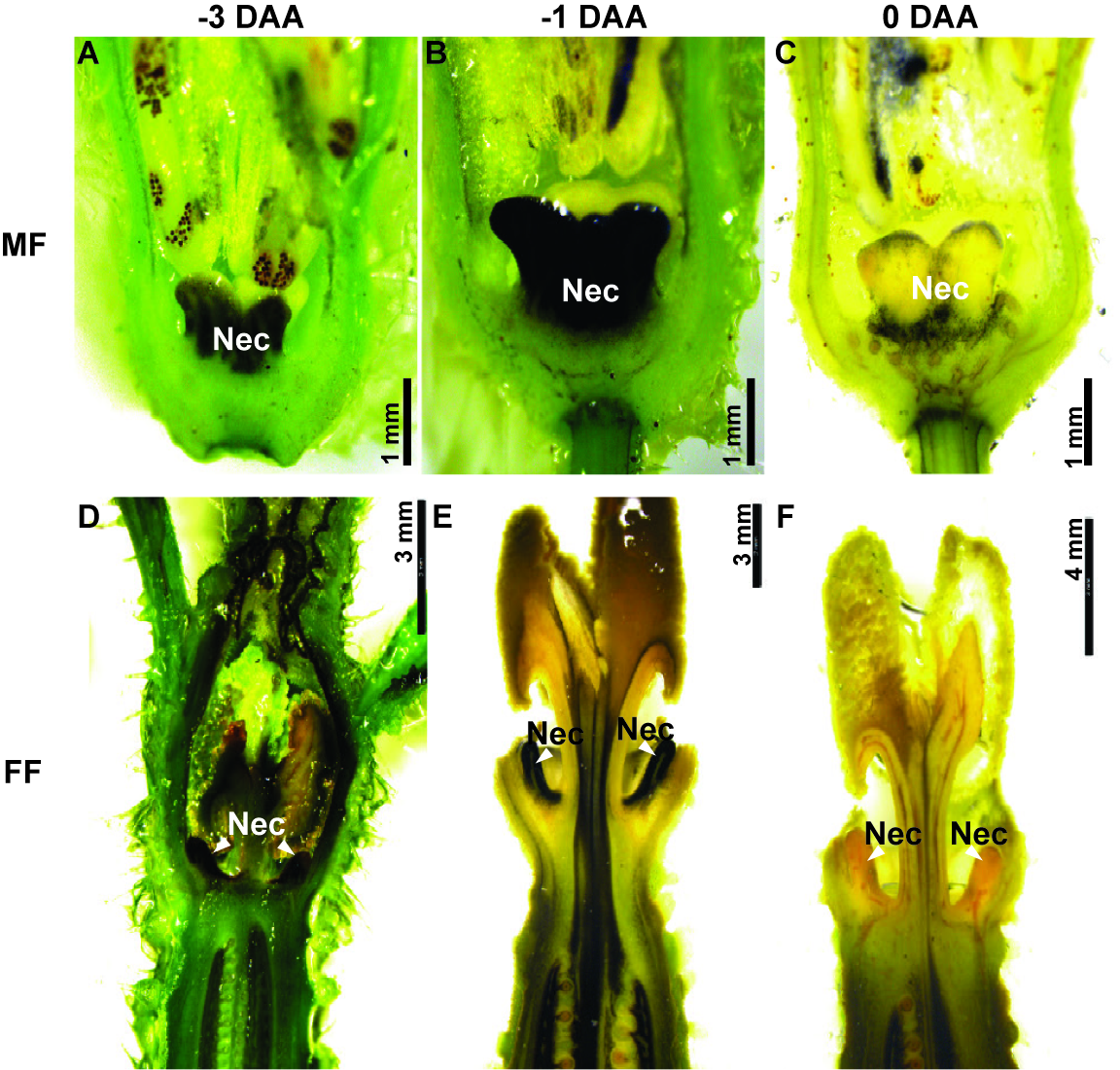

Supplement: Supplementary Figure 3 — Starch staining in nectary of male and female cucumber flowers. Different developmental stages of male (A–C) and female flowers (D–F). (A,D) is 3 days before anthesis, (B,E) is 1 day before anthesis, and (C,F) is at the day of anthesis. The white arrows in panels (D–F) indicate nectary. DAA, days after anthesis; MF, male flower; FF, female flower; Nec, nectary. [file Image_3.TIF]
